# Anti-Inflammatory Activity of *N*-Docosahexaenoylethanolamine and *N*-Eicosapentaenoylethanolamine in a Mouse Model of Lipopolysaccharide-Induced Neuroinflammation

**DOI:** 10.3390/ijms221910728

**Published:** 2021-10-03

**Authors:** Anna Tyrtyshnaia, Sophia Konovalova, Anatoly Bondar, Ekaterina Ermolenko, Ruslan Sultanov, Igor Manzhulo

**Affiliations:** A.V. Zhirmunsky National Scientific Center of Marine Biology, Far Eastern Branch, Russian Academy of Sciences, 690041 Vladivostok, Russia; sofanasrew@gmail.com (S.K.); bondar.av@dvfu.ru (A.B.); ecrire_711@mail.ru (E.E.); sultanovruslan90@yandex.ru (R.S.); i-manzhulo@bk.ru (I.M.)

**Keywords:** *N*-docosahexaenoylethanolamine, *N*-eicosapentaenoylethanolamine, synaptamide, EPEA, neuroinflammation, hippocampus, lipopolysaccharide (LPS), long-term potentiation

## Abstract

The search for methods of cognitive impairment treatment and prevention in neurological and neurodegenerative diseases is an urgent task of modern neurobiology. It is now known that various diseases, accompanied by dementia, exhibit a pronounced neuroinflammation. Considering the significant docosahexaenoic and eicosapentaenoic polyunsaturated fatty acids’ therapeutic potential, we decided to investigate and compare anti-inflammatory activity of their *N*-acylethanolamine derivatives. As a result, we found that both *N*-docosahexaenoylethanolamine (synaptamide) and *N*-eicosapentaenoylethanolamine (EPEA) prevents an LPS-mediated increase in the proinflammatory cytokines TNF-α and IL-6 production in the SIM-A9 microglia culture. In an in vivo experiment, synaptamide reversed an increase in LPS-mediated hippocampal TNF-α and IL-1β, but EPEA did not. However, both compounds contributed to the microglia polarization towards the M2-phenotype. Synaptamide, rather than EPEA, inhibited the Iba-1-positive microglia staining area increase. However, both synaptamide and EPEA prevented the LPS-mediated astrogliosis. A study of BDNF immunoreactivity showed that synaptamide, but not EPEA, reversed an LPS-mediated decrease in BDNF production. Despite the more pronounced anti-inflammatory activity of synaptamide, both compounds were effective in maintaining a normal level of hippocampal long-term potentiation in neuroinflammation. The results indicate a high therapeutic potential for both compounds. However, some tests have shown higher activity of synaptamide compared to EPEA.

## 1. Introduction

According to the World Health Organization, around 50 million people worldwide suffer from dementia. Moreover, among the population over 60 years of age, the prevalence of dementia is 5–8%. Among the diseases that cause dementia, in addition to neurodegenerative disorders, such as Alzheimer’s disease, Parkinson’s disease, frontotemporal dementia, and Lewy body disease, there is also a mild cognitive impairment (MCI). The prevalence of MCI among the elderly is 5.13–29.9% [1]. These diseases develop through a variety of molecular mechanisms, many of which are currently poorly understood. However, it is known that all neurodegenerative and most neurological diseases are characterized by a pronounced reaction of neuroinflammation. The neuroinflammatory process involves microglia and astroglial cells, which are the most common cell types in the central nervous system. When exposed to stimuli that provoke a neuroinflammation reaction, cells undergo morphological changes and begin to secrete a whole complex of factors, including the cytokines interleukin-1β (IL-1β) [2], interleukin-6 (IL-6) [3], tumor necrosis factor-α (TNF- α) [4], chemokines [5], and reactive oxygen species [6]. Moreover, once activated, cells can remain active for weeks [7]. At the same time, microglia produce factors capable of recruiting peripheral immune cells, such as macrophages, T cells, and B cells, to the inflammation focus, which exacerbates the inflammatory process, causes its chronicity, and leads to the development of neurodegenerative diseases [8]. As a rule, neuroinflammation leads to impaired cognitive functions, namely, to a violation of the hippocampus-dependent memory tasks, which indicates the involvement of the hippocampus in the processes. For example, exposure to bacterial lipopolysaccharides, which induce a potent neuroinflammatory response, leads to a violation of contextual recognition due to the activity of the hippocampal CA3 and CA1 neural circuit activity. In addition, impaired spatial memory is observed in neuroinflammation, which is based on changes in the hippocampal NMDA and AMPA receptors expression [9]. At the cellular level, proinflammatory cytokines affect the glutamate release [10], AMPA [11], and NMDA [12,13] receptor activity, as well as long-term potentiation [14]. Inflammatory mediators and immune cells in the brain also influence cognitive function through neuroplasticity, including processes such as the growth of dendrites and axons; the formation of synapses; and associated structures, neurogenesis, and apoptosis [15].

The annual increase in the prevalence of neurodegenerative diseases accompanied by neuroinflammation requires the search for their prevention and effective approaches for treatment. There is an increasing interest in drugs that regulate the activity of microglia and control the neuroinflammatory process. Some of these promising molecules include docosahexaenoic acid (DHA), eicosapentaenoic acid (EPA), and polyunsaturated fatty acids (PUFAs) which perform important structural and metabolic functions in the brain. Some studies indicate that the biological activity of DHA and EPA derivatives is significantly higher than that of the PUFAs themselves. For example, several works are devoted to the anti-inflammatory activity of natural DHA derivatives–protectins [16], resolvins [17], and maresins [18]. Moreover, it is assumed that DHA and EPA realize their activity precisely through derivatives. This is indicated by the data of some studies, for example, in the study of Kim et al. (2011), the conversion of DHA to syntamide was observed in cell culture, and the activity of synaptamide was 10 times higher than that of DHA. The use of the FAAH inhibitor (an enzyme that breaks down fatty acids ethanolamides) increased the stimulating effect of DHA on the neurite growth and synaptogenesis processes [19]. An example of such derivatives is fatty acid ethanolamides, endogenous metabolites that are synthesized in nerve cells and perform specific functions [20]. *N*- docosahexaenoylethanolamine (synaptamide) activity aimed at neurogenesis, axonal growth, and synaptogenesis is well characterized [19,21]. A number of studies show evidence of synaptamide anti-inflammatory activity [22,23,24]. Among the studied mechanisms of the synaptamide anti-inflammatory activity, a decrease in microglial activity by cAMP/PKA signaling enchantment and suppression of Nuclear factor-kappaB (NF-κB) activation are known. In addition, the activity is realized through the GPR110 receptor, binding to the synaptamide which increases cAMP accumulation [23,25]. Endogenous synaptamide production in the brain is highly dependent on dietary omega-3 PUFAs’ intake [19,26]. In addition, the biological activity of DHA concerning neurogenesis and synaptogenesis processes is significantly enhanced by fatty acid amide hydrolase (FAAH) inhibition, an enzyme that metabolizes fatty acid ethanolamides [19,21]. This once again confirms the hypothesis that DHA biological activity is realized mainly due to metabolites. EPA ethanolamide (EPEA) has a similar, but much less studied, activity. EPEA demonstrates anti-inflammatory activity in vitro on peritoneal macrophages and adipocytes, decreasing the IL-6, NO, and MCP-1 levels [27,28]. It is generally accepted that both the synaptamide and EPEA neurotropic effects are CB-receptor-independent [20]. However, there is evidence that the anti-inflammatory activity of synaptamide and EPEA is blocked by the co-presence of CB2- and PPAR-γ receptor antagonists [27]. In addition, numerous data suggest that the synaptamide and EPEA oxidative metabolites are CB1 and CB2 agonists [29]. Given the small number of studies devoted to the EPEA anti-inflammatory activity, especially in comparison with other ethanolamides, in this work we decided to compare the synaptamide and EPEA anti-inflammatory activity in in vitro and in vivo studies.

## 2. Results

### 2.1. In Vitro Studies of Cytokines Production

**Figure 1:**
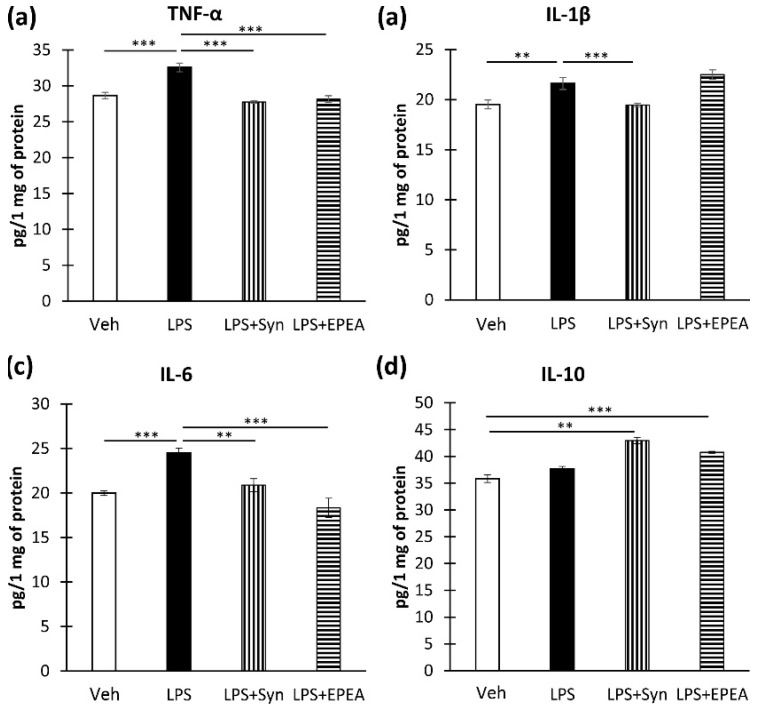
Production of cytokines in the culture of microglia SIM-A9 after LPS, synaptamide, and EPEA treatment determined by ELISA. (**a**). Production of TNF-α, pg/1 mg of protein. (**b**). Production of IL-1β, pg/1 mg of protein. (**c**). Production of IL-6β, pg/1 mg of protein. (**d**). Production of IL-10β, pg/1 mg of protein. Mean ± SEM, *n* = 5 (number of wells). Kruskal–Wallis test followed by Dunn’s multiple comparisons tests, ** *p* < 0.01, *** *p* < 0.001.

To investigate the ability of synaptamide and EPEA to suppress inflammation in vitro, we used the SIM-A9 microglia cell line. Cells preincubated with synaptamide and EPEA preparations were activated by LPS followed by ELISA. As a result, we found that synaptamide prevents LPS-mediated increase in the production of proinflammatory cytokines TNF-α (32.56 ± 0.60—“LPS” vs. 27.74 ± 0.17—“LPS + Syn”, *p* < 0.001) (Figure 1a), IL-1β (21.61 ± 0.23—“LPS” vs. 19.46 ± 0.57—“LPS + Syn”, *p* < 0.001) (Figure 1b), and IL-6 (24.48 ± 0.55—“LPS” vs. 20.88 ± 0.72—“LPS + Syn”, *p* < 0.01) (Figure 1c). EPEA also prevented an LPS-mediated increase in TNF-α (32.56 ± 0.60—“LPS” vs. 28.15 ± 0.47—“LPS + EPEA”, *p* < 0.001) (Figure 1a) and IL-6 (24.48 ± 0.55—“LPS” vs. 18.31 ± 0.09—“LPS + EPEA”, *p* < 0.001) (Figure 1d). However, EPEA was unable to reverse the LPS-mediated increase in IL-1β (Figure 1b). In addition, both synaptamide and EPEA stimulate the production of the anti-inflammatory cytokine IL-10 (35.82 ± 0.47—“Veh” vs. 42.94 ± 0.61—“LPS + Syn”, *p* < 0.01; 40.76 ± 0.17—“LPS + EPEA”, *p* < 0.001) (Figure 1d).

### 2.2. In Vivo Studies of Pro- and Anti-Inflammatory Factors Production

**Figure 2:**
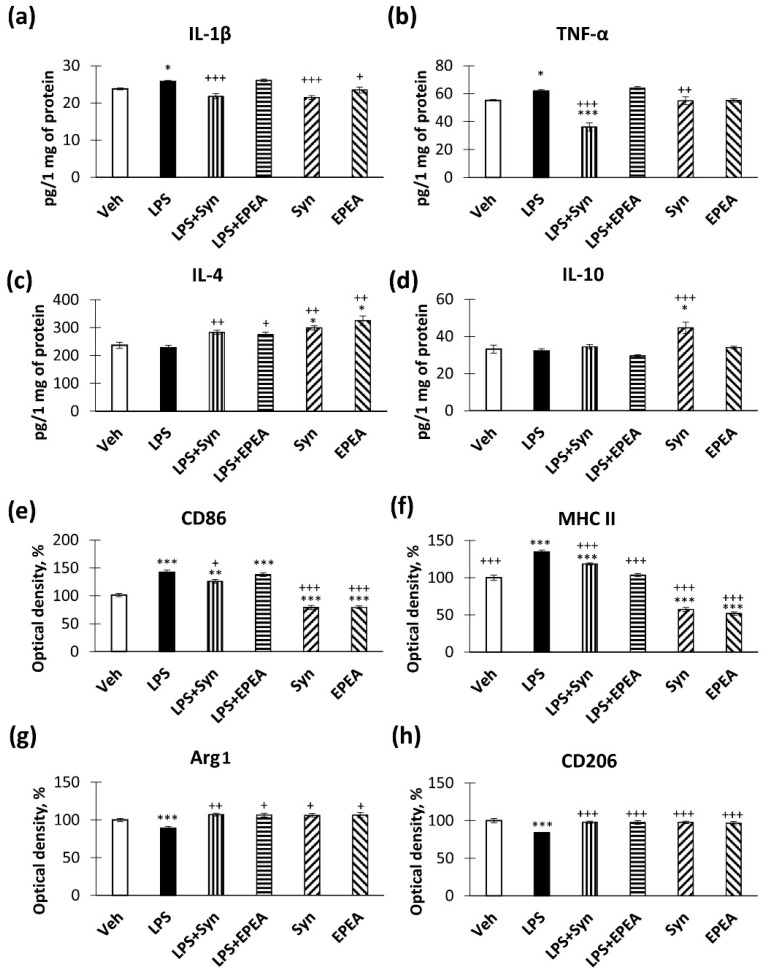
Production of pro- and anti-inflammatory factors within the hippocampus after LPS, synaptamide, and EPEA administration, determined by ELISA. (**a**) Production of TNF-α, pg/1 mg of protein. (**b**) Production of TNF-β, pg/1 mg of protein. (**c**). Production of IL-4, pg/1 mg of protein. (**d**) Production of IL-10, pg/1 mg of protein. (**e**) Production of CD86, optical density units, %. (**f**) Production of MHC II, optical density units, %. (**g**) Production of Arg1, optical density units, %. (**h**) Production of CD206, optical density units, %. Mean ± SEM, *n* = 10 (number of animals per group). One-way ANOVA with a post-hoc Tukey test, * *p* < 0.05, ** *p* < 0.01, *** *p* < 0.001; + *p* < 0.05, ++ *p* < 0.01, +++ *p* < 0.001. *—compared to Veh, +—compared to LPS.

We found that LPS causes an increase in IL-1β production (Figure 2a). The administration of synaptamide, but not EPEA, reversed the LPS-mediated increase in IL-1β production (25.78 ± 0.27 pg/mg—“LPS” vs. 21.76 ± 0.7 pg/mg—“LPS + Syn”, *p* < 0.001). A similar effect was observed in the study of TNF-α production within the hippocampus. Synaptamide reversed an LPS-mediated TNF-α increase (61.94 ± 1.00 pg/mg—“LPS” vs. 36.14 ± 2.89 pg/mg—“LPS + Syn”, *p* < 0.001). EPEA in a similar dose was unable to prevent the LPS-mediated increase in TNF-α production. Interestingly, TNF-α in the “LPS + Syn” group was lower than in the “Veh” group (Figure 2b). LPS treatment did not affect the production of the anti-inflammatory cytokine IL-4; however, synaptamide and EPEA stimulated IL-4 production compared to the control (236.56 ± 10.79 pg/mg—“Veh” vs. 299.25 ± 7.45 pg/mg—“Syn”, *p* < 0.05 and 325.18 ± 16.95—“EPEA”, *p* < 0.05) (Figure 2c). In addition, synaptamide increased IL-10 production compared to the control (33.18 ± 2.15 pg/mg—“Veh” vs. 44.60 ± 3.01 pg/mg—“Syn”, *p* < 0.001) (Figure 2d).

By studying the activity of the pro-inflammatory microglia marker CD86, we found that synaptamide, in contrast to EPEA, reduced its expression. The hippocampal CD86 level, measured by ELISA, was significantly lower in the “LPS + Syn” group (125.91 ± 3.21%) than in the “LPS” group (142.19 ± 3.68%, *p* < 0.05). In the “Syn” and “EPEA” groups, there was an even more pronounced decrease in CD86 immunoreactivity compared to the control group (101.39 ± 2.93%—“Veh” vs. 78.99 ± 3.65%—“Syn”, *p* < 0.001 and 79.45 ± 2.41%—“EPEA”, *p* < 0.001) (Figure 2e). At the same time, the production of major histocompatibility complex II (MHC II), which is also a marker of pro-inflammatory microglia, was inhibited by the administration of both synaptamide and EPEA (134.47 ± 2.78%—“LPS” vs. 118.40 ± 1.42%—“LPS + Syn”, *p* < 0.001 and 103.47 ± 2.10%—“LPS + EPEA”, *p* < 0.001). In the “Syn” and “EPEA” groups, MHCII immunoreactivity was lower than in the “Veh” group (100 ± 3.58%—“Veh” vs. 57.30 ± 2.70%—“Syn” and 51.82 ± 2.20%—“EPEA”) (Figure 2f).

In addition, we found that the compounds in the study influence the production of anti-inflammatory microglia markers. Arginase 1 (Arg1), which converts arginine to polyamines [30], and CD206, known as the mannose receptor [31], are among the most characterized anti-inflammatory M2 microglia markers. We found that exposure to LPS decreases Arg1 production, while synaptamide and EPEA prevent this decrease (88.82 ± 2.69%—“LPS” vs. 107.10 ± 1.91%—“LPS + Syn”, *p* < 0.01 and 106.47 ± 2.48%, *p* < 0.05) (Figure 2g). The situation is similar with CD206, since synaptamide and EPEA prevent an LPS-mediated decrease in this marker (83.76 ± 0.77%—“LPS” vs. 97.86 ± 1.85%—“LPS + Syn”, *p* < 0.001 and 97.55 ± 2.38%, *p* < 0.001) (Figure 2h).

### 2.3. Microglial Activity in LPS, Synaptamide and EPEA Treatment

**Figure 3:**
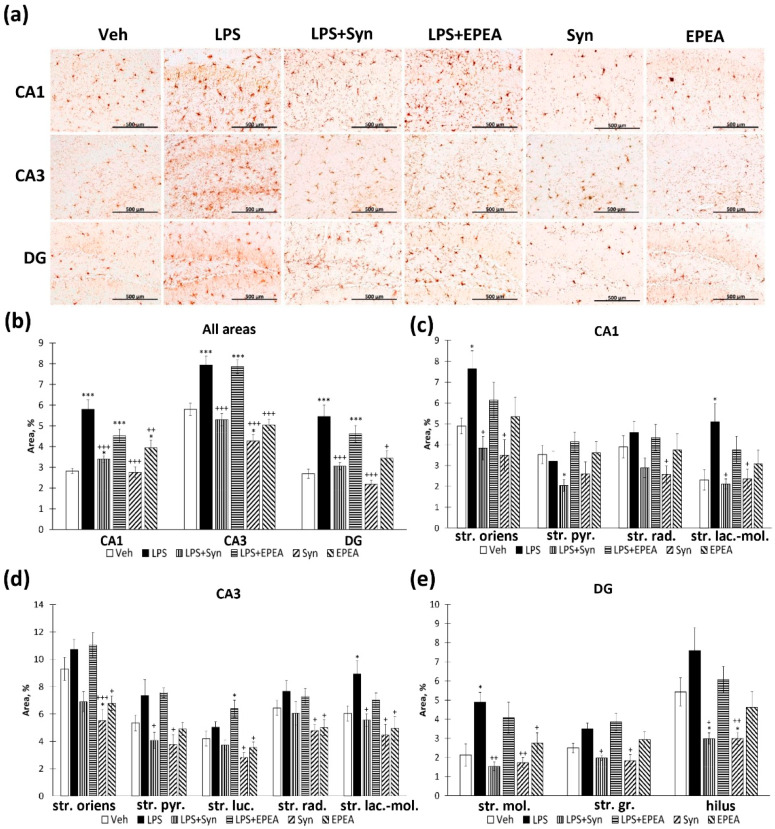
Iba-1-positive area in CA1, CA3, and DG hippocampal regions. (**a**) Representative images of Iba1-positive immunostaining in CA1, CA3, and DG hippocampal areas. Scale bar—500 µm. (**b**) Histogram demonstrating the percentage of area covered by Iba1-positive staining in CA1, CA3, and DG regions. (**c**) Histogram demonstrating the percentage of area covered by Iba1-positive staining in CA1 subregions. (**d**) Histogram demonstrating the percentage of area covered by Iba1-positive staining in CA3 subregions. (**e**) Histogram demonstrating the percentage of area covered by Iba1-positive staining in DG subregions. Mean ± SEM, *n* = 25 (number of slices per group). One-way ANOVA with a post-hoc Tukey test, * *p* < 0.05, *** *p* < 0.001; + *p* < 0.05, ++ *p* < 0.01, +++ *p* < 0.001. *—compared to Veh, +—compared to LPS. Str. oriens—stratum oriens, str. pyr.—stratum pyramidale, str. luc.—stratum luciderm, str. rad.—stratum radiatum, str. lac.-mol.—stratum lacunosum–moleculare, str. mol.—stratum moleculare, str. gr.—stratum granulosum.

We found that synaptamide prevents an increase in Iba-1-positive area staining within the hippocampus in LPS-treated animals: 5.79 ± 0.45%—“LPS” vs. 3.39 ± 0.15%—“LPS + Syn”, *p* < 0.001—CA1; 7.93 ± 0.43%—“LPS” vs. 5.29 ± 0.29%—“LPS + Syn”, *p* < 0.001—CA3; 5.44 ± 0.55%—“LPS” vs. 3.05 ± 0.17%—LPS + Syn”, *p* < 0.001—dentate gyrus (DG) (Figure 3a). At the same time, in the “LPS + EPEA” group, the values do not differ significantly from the “LPS” group (Figure 3b). It is noteworthy that, in the CA3 region, synaptamide, administered separately from LPS, reduces Iba-1 immunoreactivity compared to the “Veh” (5.79 ± 0.29%—“Veh” vs. 4.27 ± 0.30%—“Syn”, *p* < 0.05). In the CA1 (Figure 3c) and the CA3 (Figure 3d) areas, the differences in the “LPS” group are most pronounced in the stratum lacunosum–moleculare layer. In the dentate gyrus, differences are expressed in the stratum moleculare and the hilus. Synaptamide, administered separately from LPS, reduces Iba-1 immunoreactivity below the “Veh” group level within the hilus (*p* < 0.05) (Figure 3e). Such selective immunoreactivity changes are probably due to the higher microglial cells’ density in these subregions [32]. The observed heterogeneous microglia distribution within the hippocampus is probably involved in hippocampal neural activity modulating [33]. In addition, microglia often play a neuroprotective role by releasing compounds that can protect neurons from apoptotic death. The specific pattern of microglial distribution may be associated with the recruitment of microglia by factors produced by apoptotic neurons [34].

### 2.4. Astroglial Activity in LPS, Synaptamide and EPEA Treatment

**Figure 4:**
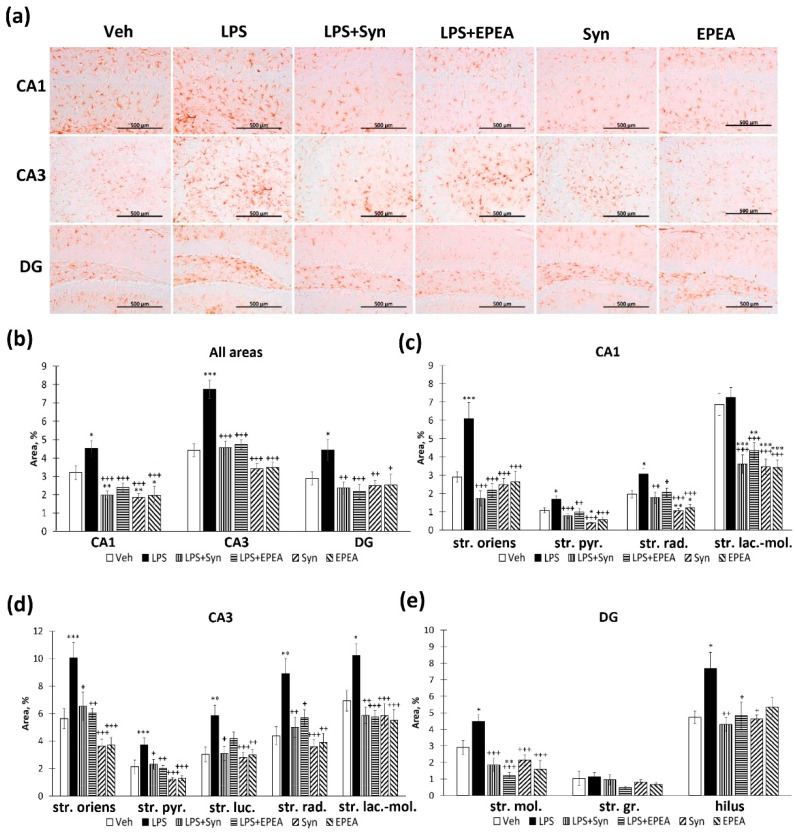
GFAP positive area in CA1, CA3, and DG hippocampal regions. (**a**) Representative images of GFAP-positive immunostaining in CA1, CA3 and DG hippocampal areas. Scale bar—500 µm. (**b**) Histogram demonstrating the percentage of area covered by GFAP-positive staining in CA1, CA3, and DG regions. (**c**) Histogram demonstrating the percentage of area covered by GFAP-positive staining in CA1 subregions. (**d**) Histogram demonstrating the percentage of area covered by GFAP-positive staining in CA3 subregions. (**e**) Histogram demonstrating the percentage of area covered by GFAP-positive staining in DG subregions. Mean ± SEM, *n* = 25 (number of slices per group). One-way ANOVA with a post-hoc Tukey test, * *p* < 0.05, ** *p* < 0.01, *** *p* < 0.001; + *p* < 0.05, ++ *p* < 0.01, +++ *p* < 0.001. *—compared to Veh, +—compared to LPS. Str. oriens—stratum oriens, str. pyr.—stratum pyramidale, str. luc.—stratum luciderm, str. rad.—stratum radiatum, str. lac.-mol.—stratum lacunosum–moleculare, str. mol.—stratum moleculare, str. gr.—stratum granulosum.

The study of the glial fibrillary acidic protein (GFAP) production within the hippocampus demonstrated a significant increase in the CA1, CA3, and DG regions (Figure 4a). At the same time, both synaptamide and EPEA prevented the LPS-mediated increase in GFAP production (CA1: 4.52 ± 0.41%—“LPS” vs. 1.97 ± 0.23%, *p* < 0.001—“LPS + Syn” and 2.39 ± 0.22%, *p* < 0.001—“LPS + EPEA”; CA3: 7.74 ± 0.50%—“LPS” vs. 4.55 ± 0.36%, *p* < 0.001—“LPS + Syn” and 4.73 ± 0.25%, *p* < 0.001—“LPS + EPEA”; DG: 4.43 ± 0.57%—“LPS” vs. 2.36 ± 0.32%, *p* < 0.01—“LPS + Syn” and 2.39 ± 0.22%, *p* < 0.001) (Figure 4b). Even though the stratum lacunosum–moleculare layer of the CA1 region does not show a significant increase in GFAP-positive staining during neuroinflammation, both synaptamide and EPEA significantly reduce the staining area compared to the control group (6.85 ± 0.60%—“Veh” vs. 3.46 ± 0.41%—“Syn”, *p* < 0.001 and 3.42 ± 0.42%—“EPEA”, *p* < 0.001). In the stratum radiatum layer, a decrease in GFAP immunoreactivity is also observed in synaptamide and EPEA-treated animals without neuroinflammation (1.96 ± 0.20%—“Veh” vs. 1.06 ± 0.11%—“LPS + Syn”, *p* < 0.01 and 1.21 ± 0.18%—“LPS + EPEA”, *p* < 0.05) (Figure 4c). In the CA3 region, both synaptamide and EPEA inhibit an increase in GFAP expression in all layers to the same extent (Figure 4d). In the dentate gyrus, a similar tendency is observed in all layers, except for the granular cells layer, where no significant differences were found between the groups (Figure 4e).

**Figure 5:**
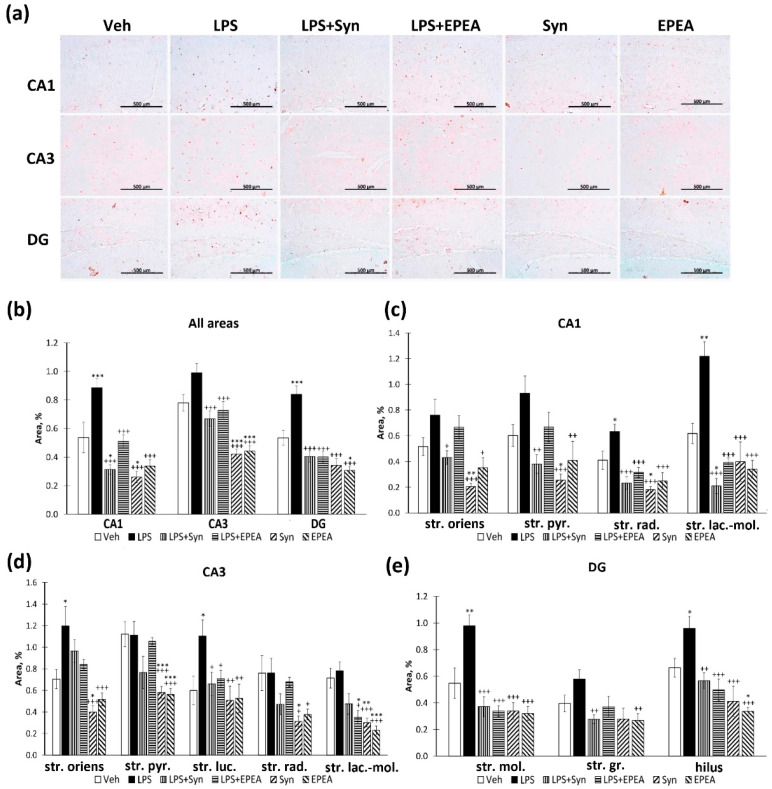
S100β positive area in CA1, CA3, and DG hippocampal regions. (**a**) Representative images of S100β-positive immunostaining in CA1 and DG hippocampal areas. Scale bar—500 µm. (**b**) Histogram demonstrating the percentage of area covered by S100β-positive staining in CA1, CA3 and DG regions. (**c**) Histogram demonstrating the percentage of area covered by S100β-positive staining in CA1 subregions. (**d**) Histogram demonstrating the percentage of area covered by S100β-positive staining in CA3 subregions. (**e**) Histogram demonstrating the percentage of area covered by S100β-positive staining in DG subregions. Mean ± SEM, *n* = 25 (number of slices per group). One-way ANOVA with a post-hoc Tukey test, * *p* < 0.05, ** *p* < 0.01, *** *p* < 0.001; + *p* < 0.05, ++ *p* < 0.01, +++ *p* < 0.001. *—compared to Veh, +—compared to LPS. Str. oriens—stratum oriens, str. pyr.—stratum pyramidale, str. luc.—stratum luciderm, str. rad.—stratum radiatum, str. lac.-mol.—stratum lacunosum–moleculare, str. mol.—stratum moleculare, str. gr.—stratum granulosum.

When evaluating an immunopositive staining of S100β, the marker of mature astrocytes, we found a pattern similar to the GFAP staining distribution (Figure 5a). Both synaptamide and EPEA reverse an increase in GFAP expression in the CA1 region (0.88 ± 0.06%—“LPS” vs. 0.31 ± 0.03%, *p* < 0.001—“LPS + Syn” and 0.51 ± 0.04%, *p* < 0.001—“LPS + EPEA”) and the dentate gyrus (0.83 ± 0.05%—“LPS” vs. 0.40 ± 0.04%, *p* < 0.001—“LPS + Syn” and 0.40 ± 0.03%, *p* < 0.001—“LPS + EPEA”). It is interesting that, in the CA3 region, both synaptamide and EPEA, administered separately from LPS, reduces S100β immunoreactivity compared to the “Veh” (0.77 ± 0.06%—“Veh” vs. 0.42 ± 0.03%—“Syn”, *p* < 0.001 and 0.44 ± 0.04%—“EPEA”, *p* < 0.001). In the DG, EPEA, administered separately from the LPS, downregulated the immunoreactivity level below the “Veh” group (*p* < 0.05) (Figure 5b). In the CA1 region, the S100β immunoreactivity was significantly increased after LPS treatment in the str. radiatum and the str. Lacunosum–moleculare, that is, in areas with the highest concentration of dendrites and synapses with Schaffer’s collateral and perforating pathway fibers (Figure 5c). Moreover, in the str. Lacunosum–moleculare, synaptamide reduces the S100β-positive astroglia staining below the control group level (*p* < 0.001). In the CA3 region, while a significant increase in S100β expression was observed in the str. oriens and the str. luciderm layers, but only in str. luciderm, we observed a significant effect of both synaptamide and EPEA on S100β immunoreactivity (1.10 ± 0.15%—“LPS” vs. 0.66 ± 0.10%, *p* < 0.05—“LPS + Syn” and 0.70 ± 0.11%, *p* < 0.05—“LPS + EPEA”). In the stratum lacunosum–moleculare, both synaptamide and EPEA reduced the S100β level in animals untreated with LPS below the “Veh” group level (*p* < 0.01 for synaptamide and *p* < 0.001 for EPEA) (Figure 5d). In the dentate gyrus, both synaptamide and EPEA prevent an increase in S100β immunoreactivity within the stratum moleculare and the hilus (Figure 5e).

### 2.5. BDNF Immunoreactivity in LPS, Synaptamide and EPEA Treatment

**Figure 6:**
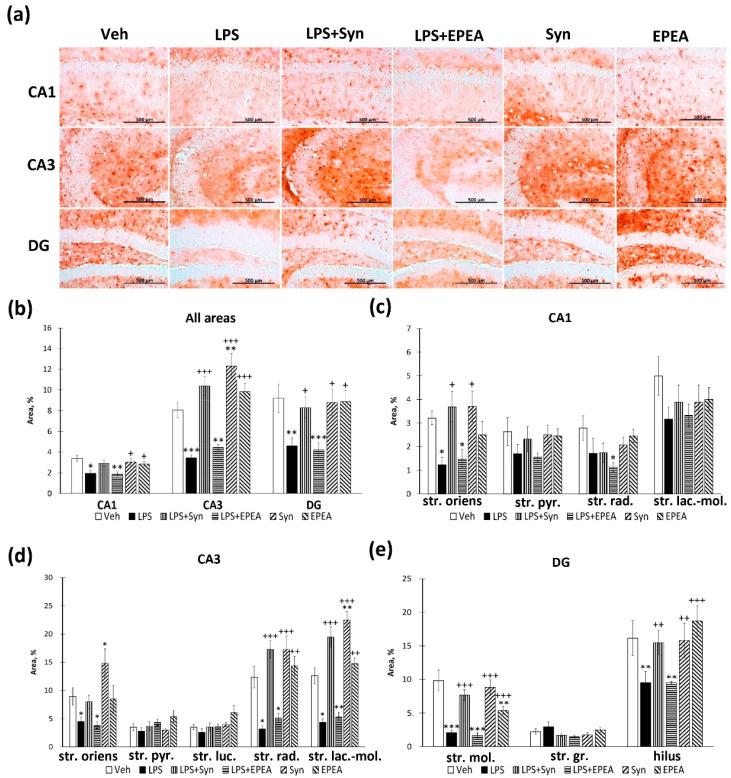
BDNF positive area in CA1, CA3, and DG hippocampal regions. (**a**) Representative images of BDNF-positive immunostaining in CA1 and DG hippocampal areas. Scale bar—500 µm. (**b**) Histogram demonstrating the percentage of area covered by BDNF-positive staining in CA1, CA3 and DG regions. (**c**) Histogram demonstrating the percentage of area covered by BDNF-positive staining in CA1 subregions. (**d**) Histogram demonstrating the percentage of area covered by BDNF-positive staining in CA3 subregions. (**e**) Histogram demonstrating the percentage of area covered by BDNF-positive staining in DG subregions. Mean ± SEM, *n* = 25 (number of slices per group). One-way ANOVA with a post-hoc Tukey test, * *p* < 0.05, ** *p* < 0.01, *** *p* < 0.001; + *p* < 0.05, ++ *p* < 0.01, +++ *p* < 0.001. *—compared to Veh, +—compared to LPS. Str. oriens—stratum oriens, str. pyr.—stratum pyramidale, str. luc.—stratum luciderm, str. rad.—stratum radiatum, str. lac.-mol.—stratum lacunosum–moleculare, str. mol.—stratum moleculare, str. gr.—stratum granulosum.

Brain-derived neurotrophic factor (BDNF) is one of the most significant regulators of brain synaptic and neurotransmitter processes [35]. As a rule, there is a significant decrease in the brain BDNF level in neuroinflammation-associated diseases [36]. Since it is known that neuroinflammation affects several signaling pathways associated with BDNF, and that glial cells are the most important BDNF source [37], we decided to investigate the level of this trophic factor in neuroinflammation and treatment with synaptamide and EPEA. We found that LPS causes a decrease in BDNF production in the CA1 (*p* < 0.05), CA3 (*p* < 0.001), and DG (*p* < 0.01) regions (Figure 6a). At the same time, in the CA3 region, synaptamide rescued an LPS-mediated decrease in BDNF production (3.42 ± 0.29%—“LPS” vs. 10.38 ± 0.89%—“LPS + Syn”, *p* < 0.05) (Figure 6b). This effect was observed mainly due to changes in the str. radiatum: 3.15 ± 0.60%—“LPS” vs. 17.29 ± 1.56%—“LPS + Syn”, *p* < 0.001 (Figure 6d). Synaptamide administered separately from LPS upregulated BDNF production within the CA3 region (8.04 ± 0.73%—“Veh” vs. 12.31 ± 1.19%—“Syn”, *p* < 0.01), which may indicate a stimulating effect of synaptamide on BDNF accumulation (Figure 6b). Similarly, synaptamide administration prevented a BDNF level decrease within the dentate gyrus (4.58 ± 0.80%—“LPS” vs. 8.27 ± 1.07%—“LPS + Syn”, *p* < 0.05) (Figure 6b). In this case, we observe changes both in the stratum moleculare (*p* < 0.001) and in the hilus (*p* < 0.01) (Figure 6e). Within the CA1 area, the changes were not so pronounced and observed only within the str. orience, where synaptamide administration reversed the BDNF level decrease (1.22 ± 0.31%—“LPS” vs. 3.68 ± 0.64%—“LPS + Syn”, *p* < 0.05) (Figure 6c). At the same time, EPEA was unable to prevent a decrease in BDNF production within the hippocampus (Figure 6b). Immunohistochemical results on hippocampal BDNF production were supplemented with ELISA data (Figure S2).

### 2.6. Synaptamide and EPEA Prevents Synaptic Plasticity Impairment

**Figure 7:**
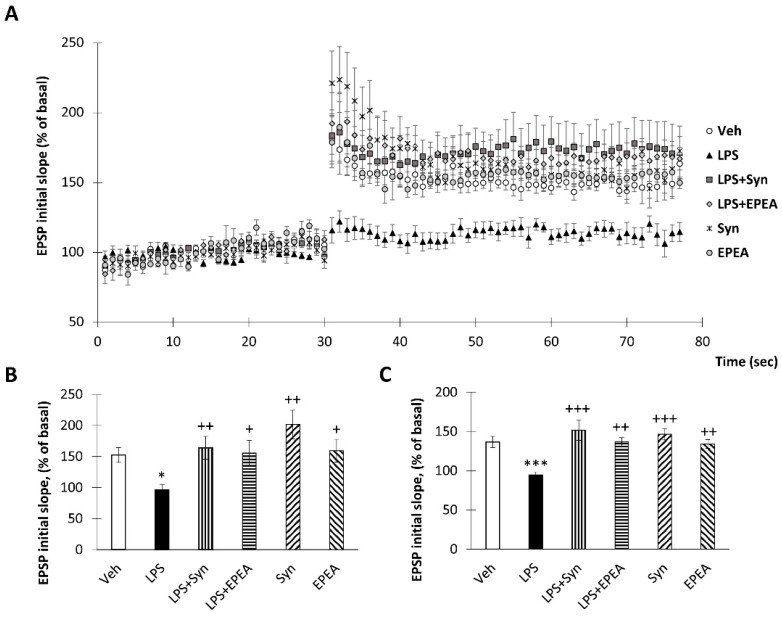
Effect of synaptamide and EPEA on LPS-induced LTP inhibition. (**a**) LPS-induced neuroinflammation suppress tetanus-induced LTP in the Schaffer collateral in mouse hippocampal slices but this effect was reversed by synaptamide and EPEA treatment. The data are expressed as the mean percentage change in population excitatory postsynaptic potential (EPSP) slope. (**b**) The averaged initial slope measured immediately after LTP, %, *n* = 5 (number of animals per group). (**c**) The averaged initial slope measured at 40 min after LTP, %, Mean ± SEM, *n* = 10 (number slices per group). The Kruskal–Wallis test followed by Dunn’s multiple comparisons tests, * *p* < 0.05, *** *p* < 0.001; + *p* < 0.05, ++ *p* < 0.01, +++ *p* < 0.001. *—compared to Veh, +—compared to LPS.

To study the effects of LPS, synaptamide, and EPEA treatment on synaptic plasticity, long-term potentiation was examined in the CA1 area of mice acute hippocampal slices. A stable baseline was recorded for 30 min before tetanic stimulation. Tetanization of the Schaffer collateral–commissural pathway induced long-term potentiation in the CA1 area (Figure 7a). The normalized field EPSPs slopes in “LPS”, “LPS+Syn” and “LPS+EPEA” groups amounted 96.04 ± 9.06% vs. 163.92 ± 18.40% (*p* < 0.05) and 172.65 ± 22.04% (*p* < 0.05) of baseline value, respectively, immediately after tetanic stimulation (Figure 7b). In 45 min after tetanization EPSPs slopes for “LPS”, “LPS+Syn” and “LPS+EPEA” were 94.42 ± 4.03% vs. 151.51 ± 12.82% (*p* < 0.001) and 136.71 ± 5.53% (*p* < 0.001), respectively (Figure 7c).

## 3. Discussion

In this study, we investigated the anti-inflammatory activity of synaptamide and EPEA in in vitro and in vivo experiments. Both studies on microglial cell culture and neuroinflammation mouse model showed an anti-inflammatory activity of the compounds. At the same time, in some tests, the activity of synaptamide was superior to that of EPEA. So, for example, in an in vitro study, EPEA, unlike synaptamide, did not restore the initial IL-1β level after LPS treatment. In in vivo experiments, EPEA was unable to reverse the increase in IL-1β and TNF-α production within the hippocampus. In contrast to synaptamide, which significantly reduced the LPS-mediated increase in Iba-1 immunoreactivity within the hippocampus, a similar dosage of EPEA did not. Although EPEA treatment did not attenuate the release of proinflammatory microglial marker CD86, a pronounced suppression of major histocompatibility complex class II expression by the microglia was observed. Both EPEA and synaptamide inhibited an LPS-mediated decrease in anti-inflammatory M2 microglia markers Arg and CD206. This may indicate that the studied substances cause microglia polarization towards the M2 anti-inflammatory phenotype. Apparently, this transformation of microglial cells underlies the prevention of LPS-induced astroglial activation. Astrocytes are the most important component of the innate and the adaptive immunity in the central nervous system, which responds to traumatic injuries and other detrimental factors [38]. This type of cell responds to various pathological influences, such as trauma, infection, ischemia, stress, etc., by activation [39]. Traditionally, astrocyte activation is thought to be primarily due to the activation of microglia, which releases a wide range of activating factors [40]. Partial M2 microglial activation with EPEA may be a key factor explaining the compounds’ ability to prevent astroglial activation without affecting the Iba1-positive microglia. The other reason for this phenomenon may lie in the impact of EPEA on an alternative pathway of astroglia activation. The Notch signaling is one such signaling pathway for astrocyte activation [41]. It was shown that LPS positively regulates the transcription of the Notch receptor ligand Jagged-1 (Jag-1), while significantly reducing the expression of the Notch-1 receptor in astrocytes [41]. This is due to the NF-κB activation through the p65/NF-κB subunit translocation into the cell nucleus. Thus, LPS probably leads to a change in astrocyte morphology by the Notch signaling blocking. EPEA may reduce the astrogliosis level due to both a decrease in the pro-inflammatory factors production by NF-kB suppression [23], and by Jag-1 and notch receptors’ expression modification [41]. At the same time, we cannot speculate about the effect of *N*-acylethanolamines on the Notch signaling in microglial cells upon their LPS activation, since this issue has hardly been studied. Based on the data that docosahexaenoic acid stimulates the Notch signaling in macrophages [42], we can assume this mechanism in activated microglial cells. Accordingly, this mechanism can serve as a potential target for *N*-acylethanolamines activity, but such assumptions require further detailed research.

Although EPEA prevented the LPS-mediated increase in astrocyte activity, it did not reverse the LPS-mediated suppression in neurotrophic factor BDNF production. Considering that astrocytes are the main source of BDNF along with neurons, the decrease in BDNF production during the development of LPS-mediated astrogliosis looks paradoxical. BDNF is involved in neuronal activity, including synaptic plasticity regulation, neurogenesis, and neuronal survival [43,44]. A decrease in BDNF levels under the influence of proinflammatory cytokines through a cAMP-dependent pathway or NF-kB has been described in previous works [45]. Reactive astrocytes are considered to be divided into two types: A1 (pro-inflammatory) and A2 (anti-inflammatory). A1 astrocytes produce pro-inflammatory factors and neurotoxins that lead to neurodegeneration and neuronal death. While A2 astrocytes promote neuronal survival and neural tissue repair [46]. It is the A2 astrocytes that produce a wide range of neurotrophic factors [46,47]. Thus, we assume that EPEA failed to prevent the polarization of astrocytes towards the A1 (pro-inflammatory) population. At the same time, synaptamide suppressed the glial activation, limiting the production of the proinflammatory cytokines, and reversed BDNF decrease.

Despite the less pronounced anti-inflammatory effect of EPEA, this substance, along with synaptamide, was able to prevent violations of synaptic plasticity within the hippocampus. We assume that this effect is due to a pronounced increase in the anti-inflammatory M2 microglial markers expression. For example, downregulation of IL-4 is known to lead to impaired long-term potentiation in the hippocampus [48], while an increase in IL-4 levels leads to impaired LTP recovery [49]. Neuroinflammation processes, as a rule, entail changes in neuronal morphology, causing cell degeneration and apoptotic death [50,51]. Considering the previously obtained data on synaptamide neuroprotective properties [19], we can assume that the tested substances reverse morphological changes in neurons and prevent their apoptotic death. However, the latter assumption requires a detailed study since no convincing data on the effect of synaptamide on apoptotic cell death have been presented yet.

Nevertheless, it is not yet clear why synaptamide and EPEA, when structurally similar, exhibit anti-inflammatory activity to varying degrees. Some previous studies also show a less pronounced biological activity of EPEA compared to synaptamide. For example, Meijerink et al. [28] showed that synaptamide is more effective than EPEA in NO release inhibiting in stimulated RAW264.7 macrophages. Furthermore, a study by Ghanbari et al. [52] showed that it is synaptamide, and not EPEA, that has an anticonvulsant effect due to the activation of the CB1 receptors. It can be assumed that EPEA has a lower affinity for CB receptors than synaptamide. However, there is still no clear position regarding CB-receptor-mediated anti-inflammatory activity. The anti-inflammatory mechanisms of synaptamide are thought to be mediated via CB-receptor-independent mechanisms [20,53]. However, some studies demonstrate that synaptamide anti-inflammatory activity is partially realized through CB2 receptor activation [29,54]. The endogenously produced synaptamide and EPEA epoxides have a pronounced affinity for the CB2 receptors through which it partially implements anti-inflammatory activity. Both synaptamide and EPEA have been shown to interact with CB1 receptors, albeit to a lesser extent than arachidonic acid ethanolamide. At the same time, the affinity of synaptamide for CB1 receptors is two times higher than that of EPEA. However, both synaptamide and EPEA activate PPAR-α receptors to almost the same extent, thus realizing anti-inflammatory activity [55]. The lower EPEA activity may also be associated with an initial low content of this compound within the brain, in contrast to synaptamide [55]. In addition, synaptamide has a lower affinity for FAAH, an enzyme that hydrolyzes N-acylethanolamines (NAEs), than EPEA, which may explain the lower tissue concentrations [54]. The fact that synaptamide, unlike EPEA, is found in blood plasma may indicate a greater degree of synaptamide involvement in metabolic processes and, accordingly, a higher activity [27,56].

In this study, we carried out a comparative analysis of *N*-docosahexaenoylethanolamine and *N*-eicosapentaenoylethanolamine anti-inflammatory activity. As a result, we demonstrated a more pronounced suppression of the proinflammatory cytokine production by synaptamide compared to EPEA in both in vitro and in vivo experiments. However, both substances suppressed the LPS-mediated decrease in M2 microglia markers. Synaptamide, in contrast to EPEA, effectively suppressed the LPS-mediated increase in Iba-1 immunoreactivity. Both compounds prevented the development of LPS-induced astrogliosis. However, the only synaptamide was found to be effective in maintaining normal levels of the neurotrophic factor BDNF within the hippocampus. Despite the lower activity of EPEA in suppressing the neuroinflammatory response, both compounds effectively prevented LTP impairment in neuroinflammation. Thus, both substances show high therapeutic potential.

## 4. Materials and Methods

### 4.1. Cell Culture

SIM-A9 mouse microglia was seeded in 24-well microplates, cultured in complete DMEM/F12 medium, and incubated at 37 °C with 5% CO_2_ for 1 h. After adhesion, the culture medium was replaced with a medium containing synaptamide or EPEA solution (10 μM) and incubated for an additional 1 h at 37 °C with 5% CO_2_. Next, an LPS solution (LPS, *E. coli* O111:B4, Sigma-Aldrich, Bellefonte, PA, USA) was added to the wells so that the final concentration was 1 μg/mL and cultured for 24 h at 37 °C with 5% CO_2_. Cells incubated in a normal culture medium without synaptamide, EPEA, and LPS were used as negative controls. As control of LPS activity, we used cells incubated in a normal culture medium without synaptamide or EPEA, but with LPS.

### 4.2. Animals and Treatments

Male C57BL/6 mice (3-month-old) were obtained from the National Scientific Center of Marine Biology, Far Eastern Branch of the Russian Academy of Sciences, Vladivostok, Russia. The mice were housed 3–4 per cage with a 12-h light/dark cycle. The animals had ad lib access to chow and water. The temperature (23 ± 2 °C) and humidity (55 ± 15%) were constant. All experimental procedures were approved by the Animal Ethics Committee at the National Scientific Center of Marine Biology, Far Eastern Branch, Russian Academy of Sciences (No 1/2021) according to the Laboratory Animal Welfare guidelines and the European Communities Council Directive 2010/63/EU.

Neuroinflammation was induced by intraperitoneal (i.p.) injections of bacterial lipopolysaccharides (LPS, *E. coli* O111:B4, Sigma-Aldrich, Bellefonte, PA, USA). Synaptamide was injected subcutaneously (s.q.) in a dose of 10 mg/kg. The mice (*n* = 80) were divided into the following treatment groups: “Veh” (*n* = 20)—i.p. saline and s.q. water injection; “LPS” (*n* = 20)—i.p. LPS and s.q. water; “LPS+Syn” (*n* = 20)—i.p. LPS and s.q. synaptamide; and “Syn” (*n* = 20)—i.p. saline and s.q. synaptamide. The i.p. saline or LPS (750 mg/kg) injections were administered for seven consecutive days. The volume of injected substances was 100 μL. The emulsion of synaptamide was prepared by mixing synaptamide with water to obtain a final concentration of 25 mg/mL with constant shaking using a Multi-Vortex shaker (V-32, Biosan, Riga, Latvia). To increase the stability of the emulsion in the process of stepwise dissolution, ethanol was added at a low concentration. For cell culture, the final concentration of ethanol did not exceed 0.1%. For in vivo administration, the amount of ethanol was 1.5% of the injected amount. A similar amount of ethanol was added to water or culture medium administered to control groups or cells.

### 4.3. N-docosahexaenoylethanolamine and N-eicosapentanoylethanolamine Preparation

**Figure 8:**
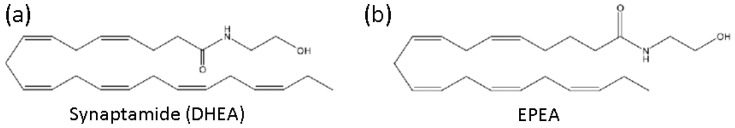
The structural formulas of synaptamide (DHEA) (**a**) and EPEA (**b**).

*N*-docosahexaenoylethanolamine and *N*-eicosapentanoylethanolamine (Figure 8) were obtained from by-products of salmon caught in the Bering Sea. The polyunsaturated fatty acid concentrate was obtained by the method of Latyshev et al. [57]. At the first stage, ethanolamines were obtained, by the conversion of a polyunsaturated fatty acid (PUFA) concentrate into ethyl esters and treatment with ethanolamine. The procedure for PUFA esterification has been described in detail earlier [57]. The reaction with ethanolamine was performed at 70 °C for at least 48 h. Then HPLC of PUFA ethanolamides was performed using a Shimadzu LC-8A chromatograph (Shimadzu, Kyoto, Japan) with UV/VIS SPD-20A (205 nm). Supelco Discovery HS C-18 preparative reverse phase column (Sigma-Aldrich, Bellefonte, PA, USA) was used for ethanolamides separation. The following parameters were used: a particle size of 10 μm, an inner diameter of 250 mm, and a length of 50 mm. We performed isocratic elution with ethanol/water (70:30, *v*/*v*). The elution rate was 50 mL/min. Fractions containing resulting *N*-acylethanolamines were collected, evaporated in vacuo, and analyzed by GC and GC-MS. The resulting *N*-docosahexaenoylethanolamine and *N-*eicosapentanoylethanolamine looked like a light-yellow oily liquids with a mild odor at room temperature. The purity of ethanolamides was 99.4%.

To determine the composition of ethanolamides, conversion to trimethylsilyl derivatives (TMS-NAE) was used [58]. For this, 50 μL of N, O-bis (trimethylsilyl) trifluoroacetamide (BSTFA) was added to 1 mg of fatty acid ethanolamides and heated to 60 °C for 1 h under argon. Then, to quantify the composition of ethanolamides , 1 mL of hexane was added, and 1 μL of each silylated fraction was injected into the GC system. A Shimadzu GC-2010 plus chromatograph with a Supelco SLB ™—5 ms capillary column 30 m × 0.25 mm inner (Sigma-Aldrich, Bellefonte, PA, USA) was used as well as a flame ionization detector (Shimadzu, Kyoto, Japan). The following conditions were applied to separate the components of the mixture: (1) an initial temperature of 180 °C; (2) a heating rate from 2 °C/min to 260 °C; and (3) the temperature was maintained for 35 min. The injector and detector temperatures were the same and amounted to 260 °C. To identify the TMS-NAE, structures GC-MS was used. Electronic impact spectra were recorded using a Shimadzu TQ-8040 instrument (Shimadzu, Kyoto, Japan) with a Supelco SLB ™—5 ms column (Sigma-Aldrich, Bellefonte, PA, USA) at 70 eV. The same temperature conditions were used as for gas chromatography. Chromatograms and mass spectra of trimethyl silyl derivates of *N*-docosahexaenoylethanolamine and *N*-eicosapentanoylethanolamine obtained by GC-MS are given in the Supplementary Materials (Figure S1).

### 4.4. ELISA

To determine the concentration of the cytokines and glial markers in the cell culture and mouse hippocampus, the enzyme-linked immunosorbent assay (ELISA) was used. For analysis, we used SIM-A9 mouse microglia cell lysate after incubation with LPS, synaptamide, and EPEA. After the cells were collected in the centrifuge tube, 0.5 mL of buffer (100 mM Tris, pH 7.4, 150 mM NaCl, 1 mM EGTA, and 1 mM EDTA; 1% Triton X-100l 0.5% sodium deoxycholate; and protease inhibitors cocktail, cOmplete™, Sigma-Aldrich, Bellefonte, PA, USA) was added to each sample. The samples (cells with buffer) were vortexed briefly and incubated on ice for 15–30 min. Then centrifuged at 13,000 rpm for 10 min at 4 °C to pellet insoluble contents. The supernatant was aliquoted to clean tubes on ice and stored at—80 °C.

The mice were anesthetized with isoflurane using rodent anesthesia vaporizer (VetFlo™, Kent Scientific Corporation, Torrington, CT, USA) and the hippocampus was quickly extracted, frozen in liquid nitrogen, and stored at a temperature of −80 °C. For analysis, we used both right and left hippocampi. The hippocampi were homogenized using a homogenization buffer consisting of 100 mM of Tris, pH 7.4, 150 mM of NaCl, 1 mM of EGTA, and 1 mM of EDTA; 1% Triton X-100; 0.5% sodium deoxycholate; and protease inhibitors cocktail (cOmplete™, Sigma-Aldrich, Bellefonte, PA, USA) incubated on ice for 15 min, centrifuged (16,000× g, 30 min, +4 °C), and the supernatants were collected. ELISA kits were used for the detection of TNF-α (ab208348), IL-1β (ab197742), IL-6 (ab46100), IL-4 (ab100710) and IL-10 (ab100697), all from Abcam, Cambridge, UK. A BCA Protein Assay Kit (Pierce, Rockford, IL, USA) was used for quantitation of protein concentration.

To determine CD86, MHCII, CD206, and Arg1 antigens, the samples (supernatants of cells or tissue lysates) were diluted with bicarbonate–carbonate coating buffer (100 mM, 3.03 g of Na_2_CO_3_, 6.0 g of NaHCO_3_, 1000 mL of distilled water, pH 9.6) to obtain a 20-μg/mL concentration. Then, 100 μL of samples (extracts from cells or tissue dissolved with coating buffer) were added to each well of PVC microtiter plate (M4561-40EA, Greiner, Austria) and incubated at 4 °C overnight. After this, the coating solution was removed, and the plate was washed three times by filling the wells with 200 µL of PBS. To block the remaining protein-binding sites in the coated wells, the 5% non-fat dry milk (M7409-1BTL, Sigma-Aldrich, St. Louis, MI, USA) was used (2 h at room temperature). After washing, 100 µL of diluted primary antibody was added to each well. In this study, we used the following primary antibodies: rabbit polyclonal anti-CD86 antibody (1:1000, ab112490), rabbit polyclonal anti-MHC class II antibody (1:1000, ab180779), rabbit polyclonal anti-mannose receptor antibody (1:1000, ab64693), rabbit polyclonal anti-liver arginase antibody (1:1000, ab96183), and rabbit monoclonal anti-BDNF antibodies (1:1000, ab108319), all from Abcam, Cambridge, UK. The plate was covered with an adhesive plastic and incubated for 2 h at room temperature. After washing, 100 µL of peroxidase secondary antibody (1:500, PI-1000-1, Vector laboratories, San Francisco, MA, USA) was added to each well, and the plate was incubated for 2 h at room temperature. After washing, 50 µL of TMB (3,3’,5,5’-tetramethylbenzidine, SK-4400, Vector laboratories, San Francisco, CA, USA) was added to each well, and the plate was incubated for 30 min at room temperature before color appears. After sufficient color was developed, 50 µL of stop solution (1N hydrochloric acid) was added to the wells.

The absorbance was measured in an iMark plate spectrophotometer (Bio-Rad, Hercules, CA, USA) at a wavelength of 450 nm. Each sample was analyzed twice, and the results were averaged.

### 4.5. Immunohistochemical Studies

Immunohistochemical studies were performed on the 7th day after the start of treatment. The animals were deeply anesthetized with isoflurane (Laboratories Karizoo, S.A., Barcelona, Spain) using a rodent anesthesia vaporizer (VetFlo ™, Kent Scientific Corporation, Torrington, CT, USA) equipped with a rodent mask. Mice were transcardially perfused with 5 mL of PBS (~4 °C), pH 7.2. Then, the brain was rapidly removed from the skull, divided into 2 hemispheres, and placed in 4% paraformaldehyde for 12 h. We used both hemispheres for immunohistochemical study. After paraformaldehyde fixation, the material was washed with PBS (pH 7.2) and embedded in paraffin blocks. After embedding in paraffin, the samples were sectioned to obtain 10-µm slices, using a Leica rotary microtome RM 2245 (Leica, Wetzlar, Germany). The immunohistochemical method used in the study consisted of the following steps: (1) blocking endogenous peroxidase activity: 0.3% H_2_O_2_ solution for 5 min; (2) blocking non-specific antibody binding: 5% BSA in PBS for 1 h; (3) primary antibodies (4 °C, 24 h); (4) secondary antibodies conjugated to horseradish peroxidase: anti-rabbit, 1:200, PI-1000-1; anti-mouse 1:200, PI-2000-1 (both from Vector Laboratories, San Francisco, CA, USA); (5) ImmPACT™ DAB Peroxidase Substrate chromogen (SK-4105, Vector Laboratories, San Francisco, CA, USA); and (6) washing with 0.1 M PBS (pH 7.2), dehydration and mounting in VectaMount Permanent Mounting Medium (H-5000, Vector Laboratories, San Francisco, CA, USA). The following primary polyclonal rabbit antibodies were used: anti-Iba-1 rabbit polyclonal antibodies (1:500, ab108539, Abcam, Cambridge, UK), anti-GFAP antibodies (1:1000, ab7260; Abcam, Cambridge, UK), anti-S100β rabbit monoclonal antibodies (1:1000, ab41548, Abcam, Cambridge, UK), and anti-BDNF rabbit monoclonal antibodies (1:1000, ab108319, Abcam, Cambridge, UK).

A Zeiss Axio Imager microscope equipped with an AxioCam 503 color and AxioVision software (Carl Zeiss, Oberkochen, Germany) was used to obtain images. The images were processed and analyzed using ImageJ software (NIH, Bethesda, MD, USA). Processing of each micrograph included the following steps: conversion to an 8-bit image; subtracting the background (rolling ball radius = 50); and contrast enhancement. To measure the area of marker staining, the necessary area was selected, and the percentage of the colored area was calculated. All measurements were performed by an operator who was blinded to the identity of the sections. For calculations, five sections were used from each animal. For statistical processing, the values obtained for each animal were averaged.

### 4.6. Electrophysiological Recordings

Mice were deeply anesthetized using isoflurane (Laboratories Karizoo, S.A., Barcelona, Spain) and decapitated; theur brains were removed and transferred to ice-cold aCSF composed of 119 mM of NaCl, 2.5 mM of KCl, 2 mM of MgCl_2_, 0.25 mM of CaCl_2_, 26 mM of NaHCO_3_, 1 mM of NaH_2_PO_4_, 10 mM of D-glucose, pH 7.4, oxygenated with carbogen 95% O_2_, 5% CO_2_. The hippocampus was removed and parasagittal sections with a thickness of 350 μm were prepared using a vibratome. The slices were allowed to recover within 1 h at 33 °C. The recordings were performed in a submersion recording chamber perfused with aCSF (30 ± 0.5 °C, 2 mL/min). To hold the slices in place in the recording chamber, we used a nylon mesh while aCSF perfusing. Acute hippocampal slices were visualized using an upright microscope (Olympus BX50). The parameters of recording extracellular electrode were: a 1.5-mm outer diameter, a length of 10 cm, and borosilicate glass (World Precision Instruments, Sarasota, FL, USA). The monopolar stimulating electrode consisted of Pl-Ir Teflon wire (75-µm diameter, including Teflon coating). The stimuli were triggered using National Instruments Labview 2019 software (10-μs duration, Master8) with an isolating stimulator (Constant Current Stimulus Isolator WPI). An intracellular amplifier in the bridge circuit mode (Axoclamp 2B, Axon Instruments), with a sampling rate of 15 Hz, was used. The signal was digitized (National Instruments, PCI 6154), analyzed, and filtered using the National Instruments Labview 2019 software.

The stimulating electrode was placed into the Schaffer collateral fiber tract between the CA2 and CA1 regions. For extracellular population excitatory postsynaptic potentials (EPSP) recording, an electrode was placed in the stratum radiatum subfield of the CA1 area at a distance of no more than 1500 μm, but not less than 300 μm from the stimulating electrode in order to avoid direct stimulation of cells located near the recording sites. To check if the slice is suitable for recording, an extra-synaptic potential was observed during stimulation of 0.5 mA, and the classic graph of input/output stimulation currents (IO) was obtained. We used a stimulation with a frequency of 1 Hz, 0.4 mA for 30 min, to stabilize the responses. For long-term post-tetanic potentiation development, the amplitude of the testing stimulus was 70% of the maximum extrasynaptic potential amplitude. Long-term potentiation (LTP) was obtained using a 100-Hz stimulation for 1 s.

### 4.7. Statistical Analysis

Data are presented as the means ± SEM. All data were tested for normal distribution using the Shapiro–Wilk test. Since the data obtained by the ELISA in in vivo experiments and immunohistochemistry were normally distributed, they were subjected to statistical analysis using one-way ANOVA followed by a post-hoc Tukey multiple comparison test. The data obtained by the electrophysiological recording and the ELISA in in vitro experiments were subjected to the Kruskal–Wallis test followed by Dunn’s multiple comparisons tests. A value of *p* < 0.05 was considered to indicate a statistically significant difference. For all studies, one animal was used as the analysis unit. All statistical tests were performed using Microsoft Excel software (Microsoft, Tulsa, OK, USA) and GraphPad Prism 4 (GraphPad Software, San Diego, CA, USA).

## Data Availability

The datasets generated during the current study are available from the corresponding author on reasonable request.

## Supplementary Materials

The following are available online at https://www.mdpi.com/article/10.3390/ijms221910728/s1.
