# Supplementary material for: Anti-Inflammatory Activity of N-Docosahexaenoylethanolamine and N-Eicosapentaenoylethanolamine in a Mouse Model of Lipopolysaccharide-Induced Neuroinflammation
Source: Int J Mol Sci. 2021 Oct 3;22(19):10728. doi: 10.3390/ijms221910728 (PMC8509568; doi:10.3390/ijms221910728)
Supplement: Supplementary file 1 [file ijms-22-10728-s001.zip › ijms-1387664-Supplementary.pdf]

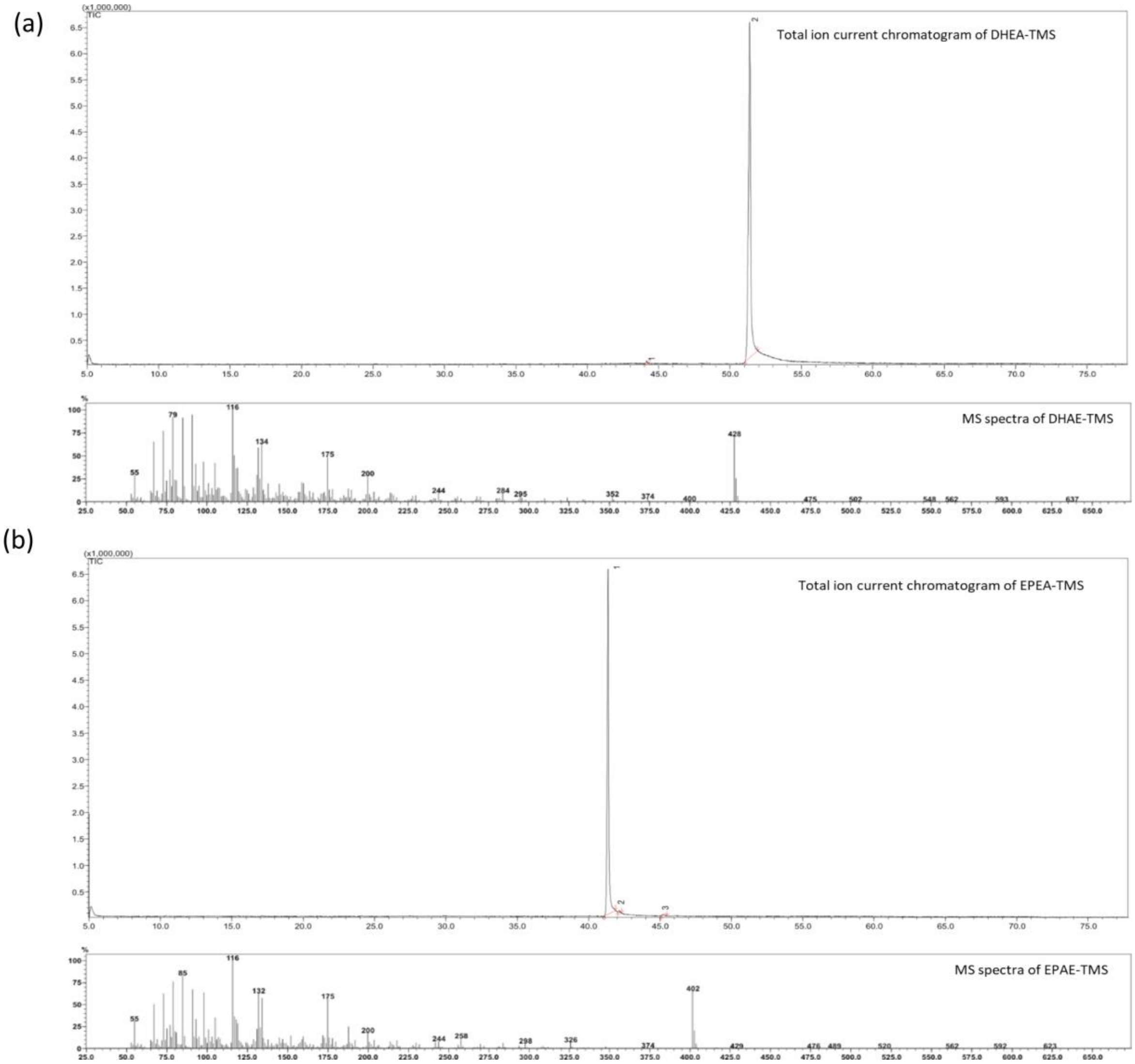

Figure S1. Chromatograms and mass spectra of trimethyl silyl derivates of *N*-docosahexaenoylethanolamine and *N*-eicosapentanoylethanolamine obtained by GC-MS.

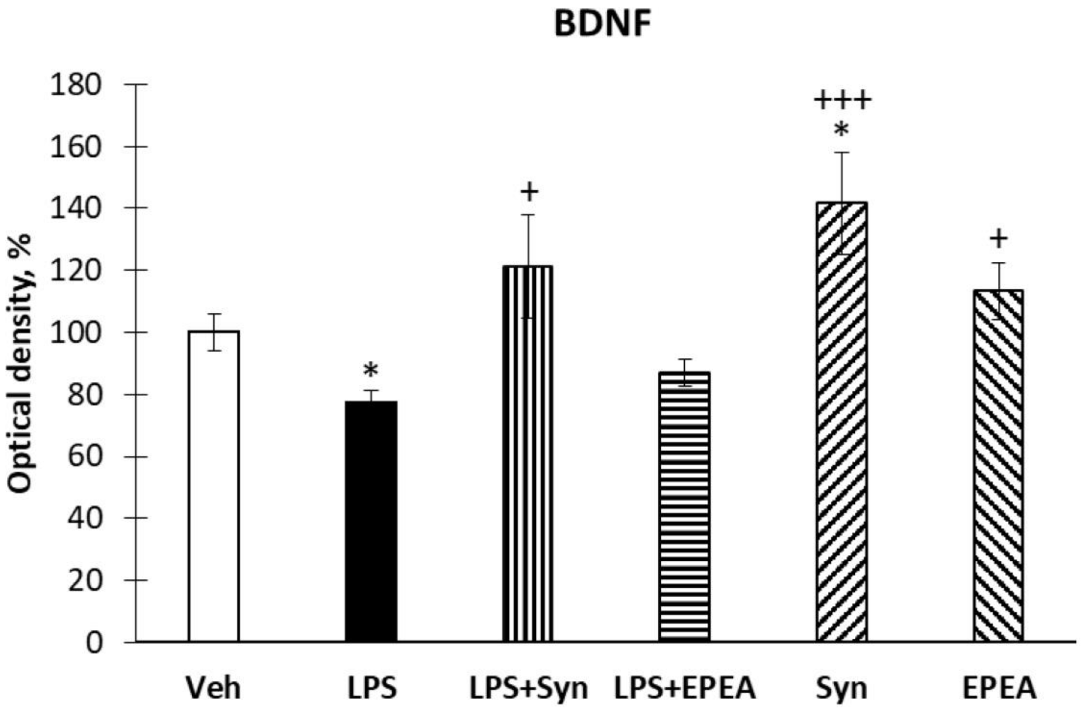

Figure S2. Production of BDNF within the hippocampus after LPS, synaptamide and EPEA administration, determined by ELISA, optical density units, %. Mean ± SEM, n = 10 (number of animals per group). One-way ANOVA with post hoc Tukey test, *p<0.05; +p<0.05, +++p<0.001. * - compared to Veh, + - compared to LPS.
